# Italian guidelines on management of persons with multimorbidity and polypharmacy

**DOI:** 10.1007/s40520-022-02094-z

**Published:** 2022-03-06

**Authors:** Graziano Onder, Davide Liborio Vetrano, Katie Palmer, Caterina Trevisan, Laura Amato, Franco Berti, Annalisa Campomori, Lucio Catalano, Andrea Corsonello, Paola Kruger, Gerardo Medea, Alessandro Nobili, Gianluca Trifirò, Simona Vecchi, Nicola Veronese, Alessandra Marengoni

**Affiliations:** 1 Department of Cardiovascular, Endocrine-Metabolic Diseases and Aging, Istituto Superiore di Sanità, Via Giano della Bella 34, 00161 Rome, Italy; 2 Aging Research Center, Department of Neurobiology, Care Sciences and Society, Karolinska Institutet and Stockholm University, Solna, Sweden; 3 Stockholm Gerontology Research Center, Stockholm, Sweden; 4 Division of Clinical Geriatrics, Department of Neurobiology, Care Sciences and Society, Karolinska Institutet, Solna, Sweden; 5 FINGERS Brain Health Institute, Stockholm, Sweden; 6 Department of Medical Sciences, University of Ferrara, Ferrara, Italy; 7 Department of Epidemiology, Lazio Regional Health Service, Rome, Italy; 8 Internal Medicine, San Camillo Forlanini Hospital, Rome, Italy; 9 Hospital Pharmacy Unit, Trento General Hospital, Autonomous Province of Trento, Trento, Italy; 10 Fondazione Policlinico Universitario “Agostino Gemelli”IRCCS, Rome, Italy; 11 Geriatric Medicine, IRCCS INRCA, 87100 Cosenza, Italy; 12 Unit of Geriatric Pharmacoepidemiology and Biostatistics, IRCCS INRCA, 87100 Cosenza, Italy; 13 European Patient’s Academy on Therapeutic Innovation (EUPATI), Rome, Italy; 14 Italian College of General Practice (SIMG), 50142 Florence, Italy; 15 Department of Health Policy, Istituto Di Ricerche Farmacologiche Mario Negri IRCCS, Via Mario Negri 2, 20156 Milan, Italy; 16 Section of Pharmacology, Department of Diagnostics and Public Health, University of Verona, Verona, Italy; 17 Geriatric Unit, Department of Internal Medicine and Geriatrics, University of Palermo, 90133 Palermo, Italy; 18 Department of Clinical and Experimental Science, University of Brescia, Brescia, Italy

**Keywords:** Multimorbidity, Polypharmacy, Frailty, Patient-centered care, Deprescribing, Models of care

## Abstract

Multimorbidity and polypharmacy are emerging health priorities and the care of persons with these conditions is complex and challenging. The aim of the present guidelines is to develop recommendations for the clinical management of persons with multimorbidity and/or polypharmacy and to provide evidence-based guidance to improve their quality of care. The recommendations have been produced in keeping with the Grading of Recommendations Assessment, Development and Evaluation (GRADE). Overall, 14 recommendations were issued, focusing on 4 thematic areas: (1.) General Principles; (2.) target population for an individualized approach to care; (3.) individualized care of patients with multimorbidity and/or polypharmacy; (4.) models of care. These recommendations support the provision of individualized care to persons with multimorbidity and/or polypharmacy as well as the prioritization of care through the identification of persons at increased risk of negative health outcomes. Given the limited available evidence, recommendations could not be issued for all the questions defined and, therefore, some aspects related to the complex care of patients with multimorbidity and/or polypharmacy could not be covered in these guidelines. This points to the need for more research in this field and evidence to improve the care of this population.

## Introduction

The progressive aging of the population has led to an increasing need for clinical guidelines and health policies to improve the management of patients with major medical, social, and care complexities [1]. The proportion of older people in the population is raising worldwide and particularly in Italy; it is estimated that more than one third of the Italian population will be aged 65 or over by 2045 [2]. Aging is associated with the accumulation of multiple chronic diseases in the same individual, a condition known as multimorbidity [3]. Multimorbidity prevalence increases dramatically after the age of 60, and most people over the age of 80 are affected by this condition [4].

One of the consequences of multimorbidity is the use of a high number of drugs, which is particularly common in older persons and defines the condition of polypharmacy. The prevalence of polypharmacy increases with age and varies between 10 and 90% in the older population [5]. In Italy, according to the national report on medicine use, 29% of older men and 30% of older women use 10 or more drugs per year [6]. In older persons, polypharmacy increases the likelihood of adverse reactions (ADR), which represents a preventable cause of unplanned hospitalization, increasing morbidity, mortality, and healthcare costs [7]. It has been estimated that approximately 5% of all hospital admissions are due to ADRs, 5% of hospitalized patients experience an ADR during their hospital stay and, 197,000 deaths per year in Europe are attributed to ADR [7]. Even if prescribing individual drugs may be recommended in disease-specific guidelines, polypharmacy is often associated with drug-drug and drug-disease interactions [8]. Therefore, both multimorbidity and polypharmacy may be associated with negative clinical outcomes, such as hospitalizations, falls, disability, and mortality [4, 9].

Overall, the above evidence suggests that the demographic transition that is resulting in an increasingly aging population needs to be supported by an adaptation of clinical practice and health and social care systems, moving from a single disease-specific approach to a person-centered approach that considers older persons’ complexities. In response to this need, in recent decades there has been a progressive evolution of the clinical and care approach to patient management, focusing on the person as a whole [10–12].

## Aims

The current guidelines aim to develop recommendations for the clinical management of persons with multimorbidity and/or polypharmacy and to provide evidence-based guidance to improve quality of care offered to persons with these conditions. The proposed recommendations are targeted to persons with multimorbidity and/or polypharmacy and their caregivers, healthcare professionals, and the healthcare system. The expected benefit of the guidelines is to improve the work of all key players involved in the patients’ care as well as to strengthen interactions between them.

## Methodology

### Expert panel

The Multimorbidity and Polypharmacy Guidelines originated from an initiative of the Italian Society of Gerontology and Geriatrics (SIGG), which involved the main national scientific societies operating in the fields of geriatrics, internal medicine, pharmacology, and general medicine, namely:

- The Italian College of General Practice (Società Italiana di Medicina Generale e delle Cure Primarie)
- The Italian Society of Internal Medicine (Società Italiana di Medicina Interna)
- The Italian Society of Hospital and Community Geriatrics (Società Italiana di Geriatria Ospedale e Territorio)
- The Italian Society of Pharmacology (Società Italiana di Farmacologia)
- The Italian Scientific Society of Hospital Internal Medicine (Federazione delle Associazioni dei Dirigenti Ospedalieri Internisti Medicina Interna)

The panel included epidemiologists, pharmacists, internists, geriatricians, pharmacologists, general practitioners, and nurses identified as representatives of the participating scientific societies. To take patient perspectives into account, in terms of values, priorities, and preferences, the panel included also a patient advocate. The patient advocate´s opinion, together with those of the whole panel, was essential for identifying welfare and organizational problems related to the management of patients with multimorbidity and polypharmacy.

The recommendations of these guidelines have been developed following the methodological manual for the production of clinical practice guidelines developed by the National Center for Clinical Excellence, Quality and Safety of Care of the Italian National Institute of Health (Centro Nazionale per l’Eccellenza Clinica, la Qualità e la Sicurezza delle Cure dell’Istituto Superiore di Sanità—v. 1.3.2April 2019), which are based on the Grading of Recommendations Assessment, Development and Evaluation (GRADE) and GRADE-Adolopment methods [13]. To improve the quality of the guidelines, two external expert referees assessed the methods and the recommendations, providing comments that were considered by the panel members in the final version of the guidelines.

### Review questions

As the National Institute for Health and Clinical Excellence (NICE) produced guidelines in 2016 on “Multimorbidity: clinical assessment and management” [12], the panel decided to use these guidelines as a conceptual frame and starting point, updating some of the review questions, and identifying new priority questions. The process of adapting the NICE recommendations and formulating new ones included several steps:

1. Evaluation and selection of review questions already addressed by the NICE guidelines that were relevant from a national perspective;
2. Identification of new review questions;
3. Definition of PICOs (Patient or Population, Intervention, Comparison and Outcome) for new review questions only;
4. Review of the literature, which included an update of the review performed by NICE researchers for questions selected from NICE guidelines or conducting a new review for each new question. When an update of NICE reviews was performed, the same search strategy adopted by NICE was used. The search was then updated by reviewing papers published after the publication of NICE guidelines.
5. Presentation of the results of the literature reviews to the panel and formulation of recommendations for each review question by members of the panel.

**Fig. 1:**
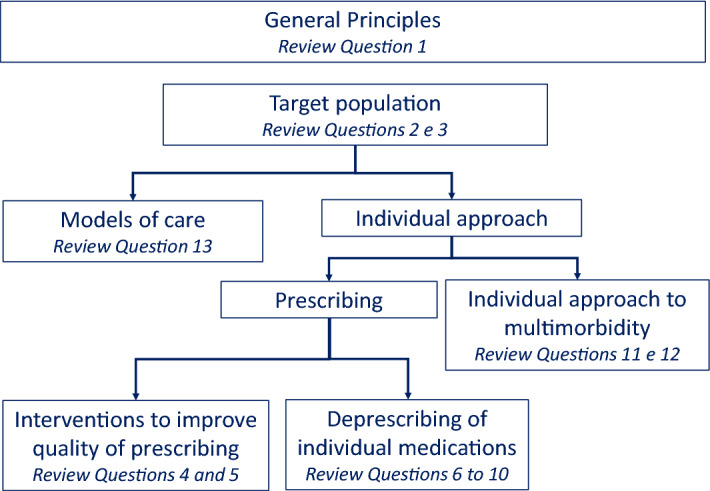
Areas covered by the guidelines and related review questions

Table 1 shows the review questions selected for the present guidelines. As described in the Fig. 1, the review questions were selected to cover several areas related to multimorbidity and polypharmacy.

**Table 1:** Review questions (Q) related to the management of patients with multimorbidity and/or polypharmacy that were defined by the expert panel

Q	Topic	Included in NICE 2016 guidelines	Type of revision
1	What principles are important for assessing, prioritising and managing care for people with multimorbidity?	✓	Qualitative
2	What risk tool best identifies people with multimorbidity who are at risk of unplanned hospital admission?	✓	Prognosis
3	What risk tool best identifies people with multimorbidity who are at risk of reduced life expectancy?	✓	Prognosis
4	Which interventions are effective for reducing polypharmacy and optimizing drug treatment?		Intervention
5	What is the clinical and cost-effectiveness of interventions to reduce polypharmacy?		Intervention
6	What is the clinical and cost-effectiveness of deprescribing antihypertensive treatment?	✓	Intervention
7	What is the clinical and cost-effectiveness of deprescribing proton pump inhibitors?		Intervention
8	What is the clinical and cost-effectiveness of deprescribing statins?	✓	Intervention
9	What is the clinical and cost-effectiveness of deprescribing aspirin or other antiplatelet drugs?		Intervention
10	What is the clinical effectiveness of vitamin D treatment in persons with multimorbidity?		Intervention
11	How effective is goal oriented care for persons with multimorbidity?		Intervention
12	What is the clinical and cost-effectiveness of self-management and expert patient programs for people with multimorbidity?	✓	Intervention
13	What models of care models improve outcomes in patients with multimorbidity?	✓	Intervention

### General principles—Question 1

Several guidelines have been proposed with the goal of issuing recommendations for assessment, prioritization, and management of care for persons with multimorbidity and polypharmacy. In previously published guidelines, recommendations are provided not only for persons with multimorbidity or polypharmacy and their caregivers, but also for healthcare professionals, and the organization of the healthcare system, with the ultimate goal of optimizing the process of care for these individuals. The panel discussed the importance of identifying the key principles that healthcare professionals should consider when assessing, prioritizing, and managing care in people with multimorbidity and polypharmacy through a review of international guidelines regarding these issues.

### Target population for an individualized approach to care—Questions 2 and 3

The panel acknowledged the fact that persons with multimorbidity are heterogeneous, and their global health status and risk of negative outcomes may vary largely. It is well known that the consequences of multimorbidity cannot be linearly estimated on the basis of the number of clinical conditions. Consequently, strategies for patients’ stratification are needed to identify the most demanding and complex-to-treat groups, i.e., those that might benefit most from individualized and integrated healthcare approaches. For this reason, the panel established that review questions should focus on the identification of tools that can support the risk-stratification of the population with multimorbidity and the identification of patients who should be targeted by the specific care approaches defined in the current guidelines.

### Individualized care of patients with multimorbidity and/or polypharmacy—Questions 4–12

The panel recognized that many persons with multimorbidity and polypharmacy have complex health needs and, as such, the standard single-disease oriented approach to treatment cannot be employed. The panel considered the importance of an individualized approach to care for these persons and recognized that the identification of health priorities and the involvement of the persons in the process of care are two pillars for tailored interventions. In addition, the panel underlined that applying single-disease oriented approaches often leads to polypharmacy and that a more personalized approach to drug utilization should be adopted in patients with complex health needs. The importance of reducing drug burden is widely recognized as a priority in persons with multimorbidity and on polypharmacy but it has proven to be difficult to achieve in clinical practice. For these reasons, the panel decided to address the issues of goal-oriented care, self-management, and deprescribing through dedicated key questions. Specific questions also concerned the available evidence on deprescribing antihypertensive drugs, proton pump inhibitors, statins, antiplatelets, as well as on the effectiveness of vitamin D (one of the most commonly prescribed drugs in Italy) in patients with multimorbidity.

### Models of care – Question 13

Persons with multimorbidity often have complex needs. The panel recognized that many models of care do not consider this level of complexity and are based on traditional single-disease oriented approaches. Consequently, persons with multimorbidity often receive fragmented care, which leads to clinical interventions that are inefficient, ineffective, and potentially harmful. Ideally, care for persons with multimorbidity should involve several healthcare providers, encompass multiple dimensions (i.e., clinical, functional, and social), and be based on robust scientific evidence. To date, not all integrated care programs for multimorbidity that are implemented in practice are based on such evidence. In addition, these programs are often heterogeneous and poorly standardized. The panel recognized an urgent need to identify which approaches are more suitable for managing persons with these conditions with the goal to provide them with high-quality care.

### Literature review

A search of the main scientific databases including PubMed, Medline, Embase, Cochrane and Epistemonikos was conducted, by focusing exclusively on patients with multimorbidity and/or polypharmacy. For review questions already addressed by the NICE guidelines, the expert panel decided to adopt the same search strategies used by NICE. For the remaining questions, search strategies were constructed using MeSH terms and free terms. For review questions that needed to be updated, the search was conducted from the date of research of the NICE guidelines 2016 [12] until 2020. No time limit was used for new review questions. For Review Question 1 only, websites of scientific relevance and of several organizations in the field of geriatrics, internal medicine, and family medicine were searched. The methodology adopted for studies selection, data extraction, assessment of study quality and summary of data is described elsewhere [14].

### From evidence to recommendations

Based on the certainty of the evidence and cost effectiveness (whenever available) of the interventions studied the panel issued two types of recommendations:

- Strong recommendation: benefits clearly outweigh (positive) risks, or vice versa (negative);
- Weak recommendation: benefits and risks are balanced or are uncertain.

For 3 review questions (Questions 6, 9, and 11), given the limited evidence available, no recommendations were issued.

## Recommendations

### General principles

*Review question 1*: What principles are important for assessing, prioritising, and managing care for people with multimorbidity?

*Recommendation.* To achieve optimal outcomes in persons with multimorbidity and/or polypharmacy, the following principles concerning the interaction of healthcare professionals and patients are recommended:

- Identify health trajectories, clinical care needs, and person preferences for their care plan.
- Agree on an individualized care plan that takes into account the interaction between chronic disease and drug treatments, as well as personal preferences about care and living environment, which include:
- defining realistic therapeutic targets and treatment plans for both the present and future (including advanced care planning);
- identifying a person responsible for care coordination;
- sharing an individualized care plan with the person, caregivers, and all healthcare professionals involved in the care process;
- scheduling regular follow-ups, with frequent drug reviews to evaluate treatment aims, needs, efficacy and safety, to decide whether to start new treatments, continue ongoing ones, and/or suspend unnecessary ones;
- establishing rules to regulate and simplify access to emergency care.
- Educate patients and/or caregivers about the use of medications and support self-management of treatment, while increasing their knowledge on the risks and benefits of polypharmacy and providing information about deprescribing procedures.

*Strength of the recommendation:* Strong.

*Recommendation.* To implement an optimal approach to patients with multimorbidity and/or polypharmacy, healthcare professionals should consider the following principles:

1. Contextualize the scientific evidence.
2. Assess the benefit/risk ratio of using specific guidelines for single diseases, in light of the patients´ clinical and social care characteristics and personal preferences.
3. Use drugs with documented efficacy, at the minimum effective dose, with the lowest number of dosage units and daily administrations.
4. Look out for adverse drug reactions due to drug interactions (including drug-drug, drug-disease, drug-food, drug-dietary supplement interactions), potentially inappropriate prescriptions, and prescriptive cascades, by applying drug prescription appropriateness criteria and/or using computerized prescription support tools.

*Strength of the recommendation:* Strong.

*Recommendation.* Care pathways for persons with multimorbidity and/or polypharmacy should be organized according to the following principles:

1. Improve coordination and collaboration between healthcare professionals and social workers and between hospital and community care, as well as integrating and promoting continuity of care.
2. Develop and use effective technologies and systems for sharing information between social and healthcare services.
3. Promote professional education and training on the topics of multimorbidity and polypharmacy, as well as measures to prevent chronic diseases.

These principles should be incorporated into national strategy plans for research on multimorbidity, polypharmacy, and deprescribing. Current guidelines should be updated to reflect these issues and implementation processes should be launched.

*Strength of the recommendation:* Strong.

### Target population for an individualized approach to care

*Review question 2:* What risk tool best identifies people with multimorbidity who are at risk of unplanned hospital admission?

*Recommendation.* The Frailty Index can be used to identify persons with multimorbidity at risk of unplanned hospital admissions.

*Strength of the recommendation:* Weak.

*Review question 3:* What risk tool best identifies people with multimorbidity who are at risk of reduced life expectancy?

*Recommendation. *Among patients hospitalized or discharged from hospital, validated tools such as the Clinical Frailty Scale (CFS), Frailty Index, and Multidimensional Prognostic Index (MPI) are recommended for identifying those with multimorbidity and limited life expectancy.

*Strength of the recommendation:* Strong.

*Recommendation.* In community-dwelling persons, the Charlson Comorbidity Index, Frailty Index, and gait speed test can be used to identify those with multimorbidity and limited life expectancy.

*Strength of the recommendation:* Weak.

### Individualized approaches to care of patients with multimorbidity and/or polypharmacy

*Review question 4:* Which interventions are effective for reducing polypharmacy and optimizing drug treatment?

*Recommendation.* Interventions to reduce polypharmacy and optimize drug treatment must be based on a comprehensive, multidimensional assessment with, whenever possible, a multidisciplinary approach, active involvement of the person and/or caregivers, and identification of inappropriate prescribing through standard criteria and/or the use of digital support tools for deprescribing. It is essential to follow the patient up to assess compliance with any intervention that has been initiated, and to detect and manage deprescription-related symptoms.

*Strength of the recommendation:* Strong.

*Review question 5:* What is the clinical and cost-effectiveness of interventions to reduce polypharmacy?

*Recommendation.* Interventions to reduce the number of drugs and optimize drug treatment are recommended to reduce the risk of falls in older persons with multimorbidity and/or polypharmacy. Such interventions should be based on a comprehensive assessment of the patient, preferably using a multidisciplinary approach, the assessment of inappropriate prescribing using standard criteria and/or digital tools to support deprescribing, the estimation of cumulative drug toxicity, the assessment of fall risk, and the active involvement of the patient and/or caregiver.

*Strength of the recommendation:* Strong.

*Review questions 6:* What is the clinical and cost-effectiveness of deprescribing antihypertensive treatment?

### Panel note to review Question 6

Low- to medium-quality evidence suggests that deprescription of antihypertensive treatment is noninferior to standard clinical practice in blood pressure management. However, the available evidence does not examine long-term clinical outcomes and provides heterogeneous results. Therefore, it is not possible to provide recommendations regarding antihypertensive treatment discontinuation. The panel emphasizes the need for further studies to evaluate the cost-effectiveness of antihypertensive treatment deprescribing in persons with multimorbidity or polypharmacy.

*Review question 7:* What is the clinical and cost-effectiveness of deprescribing proton pump inhibitors?

*Recommendation.* Deprescribing of proton pump inhibitors is recommended in persons who do not have a clear indication for these drugs (see indications of the Italian Medicines Agency in the *Appendix*).

Proton pump inhibitors should be deprescribed after a maximum treatment period of 6 weeks in persons who are treated for pyrosis, dyspepsia, or other symptoms of gastroesophageal reflux disease (in the absence of Barrett's oesophagus, severe esophagitis (grade C or D), documented history of gastrointestinal bleeding or other therapeutic indications such as long-term use of low-dose NSAIDs or aspirin in those at high-risk of bleeding) who have experienced an improvement in symptoms. New treatment with proton pump inhibitors may be considered if symptoms flare-up (i.e., on demand use).

*Strength of the recommendation:* Strong.

*Review Question 8:* What is the clinical and cost-effectiveness of deprescribing statins?

*Recommendation.* Treatment with statins as a primary and/or secondary prevention should be stopped in all persons who have a life expectancy of less than 1 year.

The decision to deprescribe statins in patients over 80 years of age who are using them as a primary prevention should be based on:

1. evaluation of the risk–benefit of the treatment (in terms of cardiovascular risk factors, life expectancy, frailty, and drug-drug interactions);
2. discussion with the person and shared decision making on the therapeutic options.

*Strength of the recommendation:* Strong.

*Review question 9:* What is the clinical and cost-effectiveness of deprescribing aspirin or other antiplatelet drugs?

### Panel note to review Question 9

The limited availability of evidence and the low quality of the only available study on this topic means that no recommendations can be made. Generation of new evidence through intervention studies is needed.

*Review Question 10:* What is the clinical effectiveness of vitamin D treatment in persons with multimorbidity?

*Recommendation.* Regardless of the patient´s plasma 25(OH)D levels, Vitamin D treatment is only recommended for older patients with multimorbidity and osteoporosis to reduce fracture risk or in institutionalized patients to reduce the risk of falls.

*Strength of recommendation:* Weak.

*Recommendation.* Regardless of the patient´s plasma 25(OH)D levels, Vitamin D treatment in older persons with multimorbidity should not be used for the prevention or treatment of conditions other than osteoporosis or falls (e.g., infections, cardiovascular diseases, and cancer).

*Strength of the recommendation:* Weak.

*Review question 11:* How effective is goal-oriented care for patients with multimorbidity?

### Panel note to review Question 11

The panel acknowledges the limitations of the studies available on this topic and the lack of substantial differences between the experimental interventions and the control groups focusing on this topic. Therefore, the panel cannot issue any recommendations, but underline an urgent need to improve the quality of research in the area of goal-oriented care. This research should take into account the following issues:

- finding a consensus on a working definition of the model of goal-oriented care and on an appropriate methodology to assess the accuracy of the model;
- comparing the model with control groups assigned to standard treatments that do not include characteristic elements of the experimental intervention;
- defining outcome indicators relevant to patients and caregivers, assessed with validated tools.

*Review Question 12:* What is the clinical- and cost-effectiveness of self-management and expert patient programs for people with multimorbidity?

*Recommendation.* To increase self-management of diseases and related treatment as well as improve quality of life, persons with multimorbidity and their caregivers should be considered for educational interventions that use multidisciplinary and personalized approaches involving healthcare professionals, caregivers, and expert patients.

*Strength of the recommendation:* Weak.

### Models of care

*Review question 13:* What models of care improve outcomes in patients with multimorbidity?

*Recommendation.* To improve health outcomes in persons with multimorbidity and polypharmacy, care models with the following features are recommended: comprehensive assessment, multidisciplinary approach, active patient involvement in care choices, individualized treatment plans, and regular follow-up.

*Strength of the recommendation:* Strong.

## Conclusion

The present guidelines were produced with the involvement of the most relevant Italian scientific societies working in the field of aging, general and internal medicine, and pharmacology with the aim to improve the quality of care and to provide guidance to healthcare professionals who are involved in the management of persons with multimorbidity and/or polypharmacy [14]. The recommendations can support the provision of individualized care to people with multimorbidity and/or polypharmacy as well as the prioritization of care through the identification of individuals at increased risk of negative health outcomes. In addition, they underline the importance of sharing care decision with patients by their involvement in the development of individualized care plans that take into account individual preferences and care goals and by their continuous educations about the use of medications and support self-management of treatments. However, not all aspects related to the complex care of persons with multimorbidity and/or polypharmacy are covered in these guidelines and, given the limited available evidence, recommendations could not be issued for all the review questions defined. This underlines the need for more research to improve knowledge that can lead to better care in this population.

## Appendix

Full text of the guideline is available at https://snlg.iss.it/wp-content/uploads/2021/10/LG-314-SIGG_multimorbilit%C3%A0-e-polifarmacoterapia_rev3.pdf

Indications for use of Proton Pump Inhibitors defined by the Italian Medicines Agency.

Prevention of serious upper gastrointestinal tract complications in patients receiving chronic treatment with non-steroidal anti-inflammatory drugs (NSAIDs) or antiplatelet therapy with low-dose ASA if one of the following risk conditions is present:

- history of previous digestive bleeding or peptic ulcer
- concomitant therapy with anticoagulants or corticosteroids
- advanced age.

Duration of treatment 4 weeks (occasionally 6 weeks) if one of these conditions is present:

- duodenal or gastric ulcer positive for Helicobacter pylori (H. pylori)
- for 1 or 2 weeks in combination with drugs eradicating H. pylori infection
- negative duodenal or gastric ulcer (first episode)
- gastroesophageal reflux disease with or without esophagitis (first episode)

Longer duration of treatment (to be reassessed after 1 year) if one of these conditions is present:

- Zollinger-Ellison syndrome
- relapsed H. pylori-negative duodenal or gastric ulcer

Gastroesophageal reflux disease with or without esophagitis (relapsing).
